# Experiences, Preferences, and Needs of Adolescents and Urban Youth in Contraceptive Use in Conakry, 2019, Guinea

**DOI:** 10.3389/fgwh.2021.655920

**Published:** 2021-08-05

**Authors:** Charlotte Bangoura, Nafissatou Dioubaté, Hawa Manet, Bienvenu Salim Camara, Mariama Kouyaté, Moussa Douno, Moses Tetui, Alison M. El Ayadi, Alexandre Delamou

**Affiliations:** ^1^Maferinyah National Centre for Training and Research in Rural Health (CNFRSR), Forécariah, Guinea; ^2^Africa Centre of Excellence for Prevention and Control of Transmissible Diseases (CEA-PCMT), University Gamal Abdel Nasser, Conakry, Guinea; ^3^School of Pharmacy, Waterloo University, Waterloo, ON, Canada; ^4^Department of Epidemiology and Global Health, Umeå University, Umeå, Sweden; ^5^Department of Health Policy, Planning, and Management, Makerere University School of Public Health, New Mulago Hospital Complex, Kampala, Uganda; ^6^Department of Obstetrics, Gynecology, and Reproductive Sciences, University of California, San Francisco, San Francisco, CA, United States

**Keywords:** experiences, preferences, needs, adolescents, youth, urban, contraceptives, Guinea

## Abstract

**Introduction:** The use of contraceptive methods is very low in Guinea, particularly among adolescents and young people. The purpose of this study is to analyze the experiences and expectations of adolescents and young people regarding the use of contraceptive methods in 2019 in Conakry, Guinea.

**Methods:** We conducted a 6-month qualitative and descriptive study. Data were collected through individual in-depth interviews and focus group discussions with adolescents and young people, health providers and health policy makers. Two approaches of deductive and inductive analysis were used to synthesize the main insights from the data.

**Findings:** Twenty-six participants were included in this study. Adolescents and young people have personal, family and community experiences that positively or negatively influence their contraceptive needs and preferences. Positive experiences include the relative cost of injectable forms, perceived absence of side effects of implants, proven efficacy and duration of action of the modern method used (implants and injectable form). Negative experiences included cost of implants remain high (15 Euros), perceived side effects including weight gain, pill compliance, method indiscretion, and low sensation of sexual pleasure for the condom. The preferences of the young participants were dominated by Implants and injectable forms that better meet their contraceptive needs. In terms of needs, the expectations expressed revolved around needs related to the health system, including sex education, reduction in the cost of some contraceptives (implants), availability of contraceptive methods, and equity in the provision of family planning services to adolescents and young people.

**Conclusion:** Exploring the contraceptive experiences, needs and preferences of adolescents and young people reveals decision-making dilemmas. Adolescents and young people expressed their experiences in terms of the cost of preferred contraceptives (implants), side effects, proven efficacy, and duration of action. However, their decisions are still influenced by availability, equity in service delivery, and the involvement of parents and religious leaders in sex education. Decision-makers should then place particular emphasis on improving health service delivery, adolescent sexual and reproductive health, availability of preferred contraceptive methods at affordable cost, and a program on sexuality education with the involvement of parents and religious leaders and the promotion of condom use.

## Introduction

In resource-constrained countries, about 10 million unintended pregnancies occur each year among adolescents aged 15–19 in low contraceptive prevalence settings (1). Improved access to family planning (FP) information and services can reduce the number of teenage pregnancies and early deliveries, as well as the number of deaths from resulting complications (2, 3). Matching FP services to the needs and preferences of adolescents and young people can improve their reproductive health, particularly by increasing the use of contraceptive methods (2, 4, 5).

In sub-Saharan Africa, adolescents and young people have a variety of experiences that influence their preferences and use of contraceptive methods. These experiences and preferences relate to the type and geographic accessibility of health facilities, frequency of service delivery, provider attitudes, confidentiality of services, waiting time at the health facility, availability, diversity, and cost of contraceptive products, cultural and religious norms of communities, and gender (6–10). Thus, special attention needs to be paid to these aspects in order to effectively meet the contraceptive needs of adolescents and young people.

Half of the Guinean population is between 15 and 24 years old, with 15–19 year group as adolescents and 20–24 year group as young people (11). Contraceptive prevalence among women of reproductive age (15–49 years) remains low in Guinea, although it has increased in recent years, from 9% in 2012 to 11.3% in 2018 (12, 13). Contraceptive prevalence among adolescents (10.6%) and young women (11.7%) also remains low (13).

The Guinean Ministry of Health recognizes that there are specific challenges to meeting the contraceptive needs of Guinean adolescents and young people; however, funding has largely focused on increasing the supply of family planning services, ignoring the operations research and action research needed to understand needs, stimulate demand, and improve the supply of services (13, 14).

The objective of this study is to analyze the experiences, preferences, and expectations of adolescents and young people regarding the use of contraceptive methods in Conakry, Guinea.

## Method

### Study Design

This was a descriptive qualitative study conducted in the city of Conakry (from June 1 to October 30, 2019).

### Study Framework

**Figure 1:**
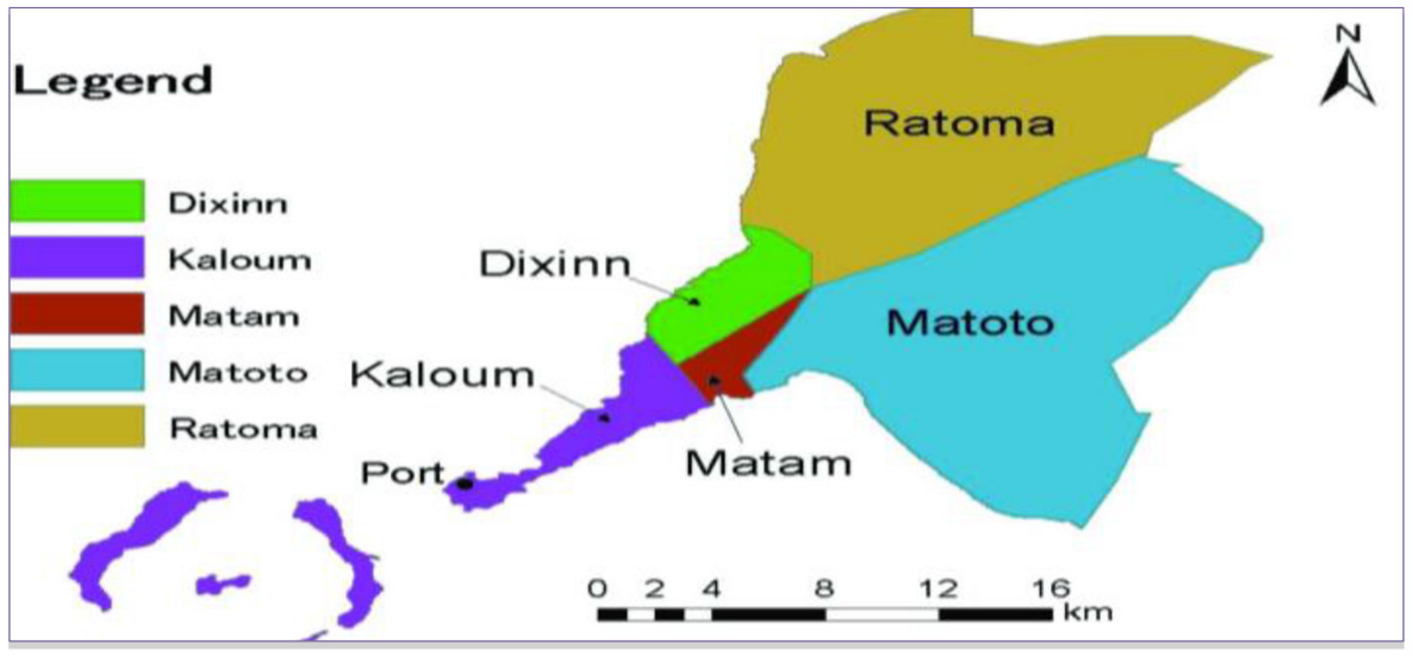
Conakry with its 5 communes according to the national direction of the territory (13).

Our study took place in the city of Conakry, and more precisely in each of its five communes (Kaloum, Dixinn, Ratoma, Matam and Matoto) (Figure 1). Conakry, the capital of Guinea, is the largest city in the country with an estimated population in 2019 of 2,317,376 inhabitants and a density of 5,150 inhabitants/km^2^. Adolescents and young people (15–24 years old) represent 55.5% of the population of Conakry (11). In 2018, contraceptive prevalence among adolescents and young people in the city of Conakry was 17.1% (13).

### Study Participants and Recruitment

The study population consisted of: adolescents and young people. These participants were selected purposively, accounting for variation in their socio-demographic characteristics (age group, sex, level of education, marital status, and commune of residence). In each commune of the city of Conakry, three neighborhoods were randomly selected for data collection; in each neighborhood, one sector was randomly selected. In each selected sector participants aged 15–19 or 20–24 were selected for interviews. To include a participant, she/he should be using a contraceptive method or have a history of contraceptive use. Any eligible person who did not consent to be included in the study was not included as participant.

Participants were reached with the help of local authorities who contacted household heads to seek participation of the adolescents and young people living with them. At a first stage, participants selected by the help of local authorities were interviewed through in-depth individual interviews (IDIs). For sample size, inclusion of participants continued until data saturation, i.e., no new information emerged from new participants.

At a second stage, IDI participants participated in focus group discussions (FGDs) with additional peers whom they contacted through a snow ball sampling. Two FGDs were organized per commune of residence, that is 10 FGDs in total. FGDs were categorized into married, single, literate and illiterate participants. In total, 72 people participated in the FGDs. FGDs were facilitated by the first author (CB) with the second author (ND) as note-taker. The rationale of conducting both (IDIs) and FGDs was to triangulate participants' individual and collective views on contraceptive use, which is somehow considered as taboo in most households in the research setting. Interviews were scheduled at participants' convenience.

### Data Collection and Sources

We used individual in-depth interview (IDI) and focus group discussion (FGD) techniques that encouraged interaction among participants. Data were collected by a team of experienced and trained qualitative interviewers (young public health doctors, sociologists) and took place in locations chosen by the participants. These interviews were conducted in French or in the local language (Soussou, Malinké, and Peullh) depending on the preference of the participants. All discussions and in-depth individual interviews were recorded on Dictaphone with a built-in microphone and transcribed into French.

### Profile of Respondents

Twenty-six participants were interviewed through individual in-depth interviews (Table 1). These participants were predominantly female (65.4%) and had attended formal school (61.5%). More than half of the adolescents and young people were married (53.8%). In addition to the individual interviews, 10 focus group discussions were conducted in the five communes with the participation of 80 adolescents and young people aged 15–24.

**Table 1:** Characteristics of participants in the individual in-depth interviews (IDI).

**Socio-demographic characteristics**	**Headcount (*n* = 26)**	**Percentage (%)**
**Types of participants**		
15–19	9	34.6
20–24	17	65.4
**Sex**		
Males	8	30.8
Women	18	69.2
**Residence of participants**		
Kaloum	6	23.1
Dixinn	6	23.1
Matam	4	15.4
Ratoma	5	19.2
Matoto	5	19.2
**Schooling—teens and young peoples**		
Attended formal school	16	61.5
Did not attend formal school	10	38.5
**Marital status—adolescents and young people**		
Married	14	53.8
Singles	12	46.2

### Data Analysis

The data collected were entirely transcribed into French by members of the research team, then entered and coded using Microsoft Word and Excel Windows 16. These were examined using content analysis approach to identify the main themes and sub-themes. Data were coded by two trained encoders (CB and BSC). Each of the encoders received half of the transcriptions. After familiarization with the content of each transcription, two codebooks were developed in Excel sheets separately by both encoders, using five transcriptions. These codebooks were compared for discrepancies, similarities and complementarity during two discussion meetings including the other co-authors. One consensual codebook was then adopted and guided parallel data coding by both encoders. Information pieces relevant to the research questions were highlighted in the word document and attributed a code using the “new comment” option. Highlighted information pieces were then copied and pasted in an Excel sheet along with the name and short definition of the respective codes, as well as the ID number and characteristics of the respondents. Next step, the codes were organized into themes and sub-themes accounting for similarities and differences, using the Excel sheets. Each encoder interpreted the themes/sub-themes and identified illustrative quotes. Interpretation of themes/sub-themes as well as corresponding illustrative quotes were discussed in a third meeting with all co-authors.

### Ethical Considerations

The research protocol was approved by the National Committee of Ethics for Health Research in Guinea (CNERS). Study participants were informed and gave their written consent prior to participating in the study. Assent was obtained from a parent or household decision maker of each participant below the age of 18. Interviews were conducted in private locations to ensure confidentiality. Some of the participants were minors aged 15–17 years and gave their consent. The informed consent of their parents/guardians was also obtained for participation in the study.

## Results

Participants' stories about their experiences, preferences and needs regarding the use of contraceptive methods in urban areas were clustered into three main themes with sub-categories or themes within each theme. These three themes focused on participants' experiences (personal, family and community), preferences for contraceptive methods, needs vis-à-vis the health system (sexual education, affordability, availability of contraceptive methods, equity in service provision) and needs vis-à-vis the community (involvement of parents and religious leaders).

### Adolescents' and Young People's Experiences of Contraceptive Use

#### Individual Experiences of Urban Teens and Young People

##### Positive Experiences

Adolescents' and young peoples' individual experiences with contraceptive use are summarized by the perceived characteristics of the method used, including cost, absence of side effects, proven efficacy, and duration of action.

*Some Methods Are Affordable*. Affordability of different methods depends on the financial capacity of individual respondents. While the cost of the different methods ranges from free (distribution) or 1,000 GNF (1 eurocent) for condoms to 150,000 GNF (15 euros) for implants, different respondents, depending on their financial capacity, believe that these costs are affordable. Indeed, a few testify that they have no difficulty in finding the money to buy these methods.

“*Despite the high price of anti-ball [implant], I never had a problem with money to get it, because you don't always buy it”*. (IDI, in school, adolescent, female, current user, commune of Kaloum)

*Condom Is Effective for Pregnancy and STIs Prevention*. Users of methods such as implants and injections have testified to the effectiveness of these contraceptive methods in preventing pregnancy in their homes. In addition to teenagers and young boys who reported avoiding pregnancy and sexually transmitted infections (STIs/AIDS) in their couples by using condoms, the following are some of the most common reasons why they use condoms.

“*We use condoms for a lot of things. To keep girls from getting pregnant and also that they don't get sexual diseases”* (Participant 3, in school, adolescent, male, unmarried, current user, FGD, Commune of Dixinn)

*Some Methods Have Long-Lasting Action*. In addition, the long duration of action of the method was an advantage reported by respondents who used implants or injections. For them, these methods prevented them from becoming pregnant despite having sex without a condom for the duration of the method's action, 3 months and 3 years, respectively.

“*I use the device that you put in your hand [implant], so no one will understand that I'm planned, and lasts a lot of years, because I don't go to the hospital every time*” (Participant 2, in school, young, female; married, current user, FGD, Commune of Matoto).

##### Negative Experiences

Adolescents' and young people's personal negative experiences with contraceptive methods are related to affordability, dose-related compliance, perceived side effects, method indiscretion, and low sensation of sexual pleasure.

*Some Methods Are Not Affordable*. Respondents testified about the financial constraints to accessing contraceptive methods such as implants. Indeed, they perceive this method as their preferred method because of its effectiveness and duration of action; however, they say they are unable to bear the cost, which amounts to 150,000 GNF (15 euros) for a period of 3 years.

“*I once decided to plan ahead, I went to the hospital for the Jadelle [implant], I was told it's 150,000 GNF and I didn't have any money at the moment. So I came back without planning”* (IDI, young, female, in-school, unmarried, former user,Commune of Dixinn)

*The Daily Use of Pills Is Boring*. Some of the respondents stressed that they are challenged by the demands of daily use of methods such as the pill. Indeed, they sometimes say they forget to take pills, which sometimes results in the ineffectiveness of the method, i.e., the occurrence of pregnancy.

“… *I used to use the pills [birth control pills] even that… I got pregnant, but I forgot to take my medication a lot*” (Participant 6, young, female, unmarried, former user, FGD, Commune of Matam)

*Some Methods Have Side Effects*. Respondents who had used implants, injections and condoms reported experiencing side effects after use. Weight gain and menstrual problems were cited as side effects following the use of injections and implants. In addition, two young women reported infections from their partners' use of condoms, which they believed were caused by the lubricant.

“*I have a problem, people say to use [condoms] but when my boyfriend puts them on, the oil on them gives me infections afterwards*.” (IDI, young, female, unmarried, out school, former user, Commune of Matam)

*Implant Is Not a Discrete Method*. Furthermore, implants have been cited as a method that is not discreetly used. They find that the visibility of the implant under the skin reveals their status as a contraceptive user against their will.

“*I like anti-ball [implant] but when you put it on, everyone will see the marks when you wear the straps”* (IDI, adolescent, female, in school, unmarried, former user, Commune of Matoto)

*Condom Does Not Provide Sufficient Sexual Pleasure*. Two adolescent girls and one young woman confessed that they did not feel sufficient sexual pleasure when their partners used condoms during sex.

“*Frankly, I don't feel the taste when using condoms. It spoils my appetite”* (Participant 3, adolescent, female, unmarried, in school, former user, FGD, Commune of Dixinn)

#### Family Experiences of Adolescents and Young People

The family experiences reported by participants (parents) in this study relate to the level of parental involvement in their children's sex education. Some mothers reported being involved in their daughters' sex education from puberty onward, in that they recommend contraceptive methods to their daughters. In some places, this recommendation by mothers is motivated by negative experiences that girls or their mothers have in the family. Indeed, some mothers report that they have been threatened with dismissal from their homes because their daughters had a pregnancy outside of marriage. Others testified that their daughters were fired from their homes for the same reason.

“*When I got pregnant, I was the laughing stock of the family, I was fired by my father… Immediately after giving birth, my mom put ‘Anti-ball' [implant] since it is a long-lasting method”* (IDI, adolescent, female, out school, current user, Commune of Matoto).

Another experience is the lack of communication between parents and their children about contraceptive methods. This influences adolescents' and young people's perception of method use by the lack of support from their parents, especially financial support, to purchase a contraceptive method. Indeed, they fear that by asking for such support, they will be in conflict with their parents, who, in their opinion, would consider that they started having sex too early.

Fear of parents also leads some adolescents and young people who are already sexually active to use contraceptive methods that are discreet, both to avoid getting pregnant and to allow their parents to know that they are using contraception.

#### Community Experiences of Adolescents and Young People

Community experiences relate to values and beliefs as well as family honor. A mother from the Peulh ethnic group argued that Peulh culture does not encourage the use of contraceptive methods by the girl. According to her, this is the reason why many teenage girls and young women, including her own, do not use the contraceptive method. In addition, one young woman let us understand that she does not use contraceptive methods under recommendation of the Muslim religion.

Moreover, the preservation of family honor, that is, keeping the girl's virginity until marriage through education transmitted by families, motivates some parents but also some adolescents and young people to opt for abstinence rather than use a contraceptive method that, according to them, encourages sexual activity. Another fear related to family integrity is the concern of parents to be safe from slander in the community in case their daughters become pregnant before marriage. This concern leads them to encourage their children to use contraception.

### Adolescents' and Young People's Preferences for Contraceptive Methods

#### Modern Methods

Both adolescents and young people expressed similar preferences for modern methods of contraception. For adolescents and young girls, the implant, injectable method and the morning-after pill were the preferred methods.

Respondents said they preferred the implant because of the absent or minimal side effects from its use and its long duration of action, saving users from having to see a service provider too often. Another advantage explaining the preference for the implant is that it restores the user's ability to become pregnant as soon as the method is discontinued. The injectable method is considered to be the best method for ensuring discretion, since it does not leave a mark on the body and does not require daily administration like the pills that must be kept on one's person. The preference for the morning-after pill lies in the fact that it is only taken occasionally, i.e., after the sexual act, and lasts only 72 h. Unlike other methods that have to be administered periodically and have a relatively long duration of action, the morning-after pill is not a pill.

“*For example, I already have a child, I'm not educated so I don't want to get pregnant anymore, I preferred the Jadelle [implant] because I won't go to the hospital every time”* (IDI, young, female, married, in school, current user, Commune of Matam)

However, young women and men prefer to use the male condom because of its dual purpose. According to them, this method not only causes no side effects but also helps prevent sexually transmitted diseases. A young people from Matam told us:

“*I prefer the condom because it doesn't create any problem on the body and helps me to avoid sexually transmitted diseases”*. (IDI, young, male, out school, unmarried, current user, Commune of Matam)

#### Traditional Methods

According to respondents, their preferences for traditional methods ranged from necklace, abstinence, upside-down canary, and papaya seeds. In fact, the necklace is a string of beads with different colors representing the different days of the menstrual cycle, making it possible to identify the days at risk and not at risk of pregnancy. In addition to these reasons, users of the necklace method also say they use it for its quality to guide non-literate users;

In addition, spiritual belief is a particular source of motivation for users of the reverse canary method. This method consists of turning the canary upside down on the ground after quoting a number of Koranic verses. Users say they never get pregnant while the canary is upside down.

As for papaya seeds, this method consists of drying the seeds and then using them in tablet form. They are used for its virtual free and no side effects, as explained by a young woman:

“*It is a method of my grandmother, the papaya seeds that I used for a long time and I found it effective, without [side] effects”* (IDI, Young, female, unmarried, out school, current user, Commune of Matam).

The preference for these different methods is mainly related to their affordability. In addition, according to our respondents, these methods do not cause side effects.

### Expectations Regarding Contraceptive Use

Adolescents and young people expect actions from the health system and the community.

#### Expectations From the Health System

Expectations from the health system regard sexual education, affordability and availability of contraceptive methods, fairness in service delivery.

##### Sexual Education

Adolescents and young people recommend a sexuality education program, especially in schools, since this is their own space (few adults are there) and is conducive to improving their knowledge of contraceptive methods.

“*We want that [family planning promoters] raise awareness at the family level [most people] do not go to school, so [family planning promoters] have to come to the families, to the parents of the students, to explain to them that it is not taboo to take the time to talk with their daughter*”. (FGD, young, in school, female, unmarried, current user, Commune of Matam).

##### Affordability

Adolescents and young people expect the health system or the government to facilitate financial accessibility, i.e., to offer certain methods free of charge or to set costs within their reach. For them this financial accessibility given their economic dependence on their parents.

“*We cannot ask money to our parents for this [contraceptive method]; they make it [contraceptive method] free of charge, then it will encourage us…*”. (FGD, adolescent, in school, female, unmarried, former user, Commune of Ratoma).

##### Availability of Contraceptive Methods

Adolescents and young people recommend that the health system take care to prevent stock-outs of contraceptive method in health facilities.

“*Sometimes you need a method, you go, they tell you it is out of stock. They should to all their best to have all methods with them anytime*”. (FGD, young, in school, female, unmarried, current user, Commune of Dixinn).

##### Fairness in the Service Delivery

Some adolescents and young people pointed out that in some health facilities, adults are given priority in the delivery of family planning compared to adolescents and young people. They therefore expect health facilities to prioritize adults in the same way as adolescents and young people in the delivery of services.

“*They should not consider adults more than us when we go there, if they want to encourage us to use contraceptive*”. (FGD, adolescent, in school, female, unmarried, former user, Commune of Kaloum).

#### Expectation From the Community

Some adolescents and young people emphasized the need for their parents and religious leaders to be involved in promoting contraception for adolescents and young people.

##### Parents' Involvement

According to adolescents and young people, the involvement of their parents in the promotion of contraception would be a facilitating factor for their financial accessibility to contraceptive methods, but also for the fact that they are reassured not to disagree with their parents when they use these methods.

“*We recommend that [family planning promoters] raise awareness of parents so that they involved themselves in their children' family planning*”. (Participant 7, former user, FGD, Young unmarried women, Commune of Matoto).

##### Religious Leaders' Involvement

Adolescents and young people emphasized that the recommendations of Islam prevent them from using contraceptive methods, a practice they believe compromises their compliance with religious rules. They therefore believe that the involvement of religious leaders in promoting contraception would reassure them in their religious conduct.

## Discussion

This study reveals that adolescents and young people have personal, family and community experiences that positively or negatively influence their needs and preferences for using modern contraceptive methods. This is despite the fact that we found that all participants we met were aware that the use of modern contraceptives including condoms can prevent unwanted pregnancies and sexually transmitted diseases.

Thus, these different experiences expressed by adolescents and young people highlight the dilemmas and difficulties inherent in influencing their decision making regarding the use of modern methods of contraception. Positive experiences of adolescents and young people included the relatively affordable cost of condoms and implants, perceived absence of side effects, proven efficacy and duration of action of the modern method used (implants and injectable form). In fact, these positive experiences for this group of adolescents and young people) are one of the main sources of motivation guiding their contraceptive preferences. The preference for implants by adolescents is explained by the fact that it is a long-lasting method with fewer side effects and reduces the use of health facilities for contraceptive research.

As for the negative experiences experienced by adolescents, in contrast to the financial accessibility of implants supported by a minority of adolescent girls, a majority find that the cost of implants remains high (15 Euros) despite their proven effectiveness and their longer duration of action. Negative experiences such as the financial accessibility of implants, compliance with the dosage (the pill), perceived side effects, indiscretion of the method, and the low sensation of sexual pleasure for the condom, weight gain after taking the pills were also raised by the study participants. Among the negative experiences, a minority of participants reported cases of infection as a result of the lubricants in the male condoms used by their partners. However, all participants were aware that contraceptives, including condoms, prevented unwanted pregnancies and sexually transmitted diseases.

Experiences, preferences and expectations of adolescents and young people regarding contraceptive use in Guinea have health system and socio-cultural implications that are key to increasing contraceptive prevalence in these groups.

From the experiences of adolescents and young people in Conakry, health system factors such as cost and availability of certain methods hinder contraceptive use by these groups. Indeed, in the Guinean context, these groups are economically dependent on their parents from whom they generally lack support for contraceptive use as illustrated in this study. Alleviating financial barriers for access of these groups to contraceptive methods would be useful to reduce FP unmet need within these groups. In Guinea, reproductive and Maternal health are national priorities (15), however, user fee exemption policies are only adopted for maternal care i.e., ANCs, childbirth and post-partum care (15). Priorities geared toward user fee exemption for all contraceptive methods for adolescents and young people in Guinea could help increase contraceptive coverage among this group. User fee exemption policies for maternal health has been reported to increase utilization of emergency obstetric care in rural Guinea (16). However, in Conakry region (urban area), payment of user fees for childbirth has been reported in government hospitals in a context of user fee exemption policies, with 95% of women paying for childbirth services (17). Achieving user fee exemption policy for FP with an integrated audit system of FP service provision is therefore needed in the Guinean context. Elsewhere, some studies have shown that contraceptive clients prefer to receive detailed information on contraceptive methods to help them make their decision. (18–21) and others stressed the importance of personalized contraceptive information tailored to their individual needs and preferences (20–22).

Socio-cultural factors are also very influential on adolescents and young people's contraceptive behaviors in Guinea, as shown in this study. Contraception is considered a taboo because it is related to sexuality; it is also considered socially and religiously deviant in Muslim religion which is the most dominant one in Guinea. Fear of being considered socially and religiously deviant therefore leads adolescent and young people either refrain from using use any method, or limit their choices to methods that they perceive as discrete. The influence of community and religious norms has also been reported in other studies (18, 19, 22).

In a context where contraceptive use is highly about preference given other factors such as cost and side effect, but also about availability, limitation of contraceptive choices to the perceived discrete ones constitutes an obstacle to contraceptive use. As recommended in this study by adolescents and young people, parents involvement in the promotion of contraception would be a facilitating factor for their financial accessibility to contraceptive methods but also for their social fulfillment. However, parents shifting their efforts toward contraceptive promotion in a society seen as influenced by religious prohibition regarding family planning requires involvement of religious leaders. In addition to the media and health personnel, religious and community leaders are among the main sources of information for adolescents and young people (22) that may influence their sexual life.

## Conclusion

Adolescents and young people expressed diverse positive or negative experiences in terms of the cost of some contraceptives preferred by adolescents and young people (implants, side effects, proven efficacy and duration of action of the modern method used) and preferences (implants and injectable form) to better meet their contraceptive needs. However, their contraceptive decisions are still influenced by the availability of contraceptives, equity in service delivery, and the involvement of parents and religious leaders in the sexual and reproductive health education of adolescents and young people.

Experiences, preferences and expectations of adolescents and young people regarding contraceptive use in Guinea have health system and socio-cultural implications that are key to increasing contraceptive prevalence in these groups.

Achieving user fee exemption policy for FP with an integrated audit system of FP service provision is therefore needed in the Guinean context. What's more, involvement of parents and religious leaders in the promotion of contraception would be a facilitating factor for their financial accessibility to contraceptive methods but also for their social fulfillment.

## Data Availability Statement

The original contributions presented in the study are included in the article/supplementary material, further inquiries can be directed to the corresponding author/s.

## Ethics Statement

The research protocol was approved by the National Committee of Ethics for Health Research in Guinea (CNERS). Study participants were informed and gave their written consent prior to participating in the study. Interviews were conducted in private locations to ensure confidentiality. Some of the participants were minors aged 15–17 years and gave their consent. The informed consent of their parents/guardians was also obtained for participation in the study.

## Author Contributions

BC analyzed the data and drafted the paper which was approved and commented by all authors. All authors contributed to the article and approved the submitted version.

## Conflict of Interest

The authors declare that the research was conducted in the absence of any commercial or financial relationships that could be construed as a potential conflict of interest.

## Publisher's Note

All claims expressed in this article are solely those of the authors and do not necessarily represent those of their affiliated organizations, or those of the publisher, the editors and the reviewers. Any product that may be evaluated in this article, or claim that may be made by its manufacturer, is not guaranteed or endorsed by the publisher.
